# Cutaneous plaque in adult T cell leukemia/lymphoma: A case report

**DOI:** 10.1097/MD.0000000000023491

**Published:** 2020-12-11

**Authors:** Chen Shuang Lei, Qian Jiang, Qinhua Yu, Liannv Qiu

**Affiliations:** a College of Medical Technology, Zhejiang Chinese Medical University; b Department of Clinical Laboratory, Zhejiang Provincial People's Hospital, People's Hospital of Hangzhou Medical College, Hangzhou, China.

**Keywords:** adult T cell leukemia/lymphoma, immunology, skin-first plaque

## Abstract

**Rationale:**

The rarity of adult T cell leukemia/lymphoma (ATLL) in China, coupled with its clinicopathologic mimicry of primary skin disease, poses a diagnostic challenge. The method of diagnosis and mechanism of immune regulation in ATLL are discussed in the present report.

**Patient concerns:**

A 51-year-old Chinese man was admitted to the hospital with 2-years history of systemic plaque lesions and 1-year history of left ankle joint pain.

**Diagnoses:**

The patient was diagnosed with ATLL based on the results of flow cytometry immunophenotype and human T-cell lymphotropic virus type 1 (HTLV-1) serology.

**Interventions:**

The patient received 3 cycles of cyclophosphamide, epirubicin/ vinorelbine, and dexamethasone (CHOP) chemotherapy. However, he relapsed and did not respond to epirubicin, vindesine, etoposide, dexamethasone (EPOCH) chemotherapy.

**Outcomes:**

His family discontinued the treatment and opted for hospice care.

**Lessons:**

Patch and plaque ATLL types exhibits a better survival rate, but atypical skin patches delays the diagnosis of ATLL and negatively affects the patient survival. Based on the present findings, we suggest that patients with petal-like nuclear lymphocytes in blood smears, a high CD4: CD8 ratio, and strong CD25 expression should undergo HTLV-1 serology testing.

## 1 Introduction

Adult T cell leukemia/lymphoma (ATLL) is a peripheral CD4^+^ T cell malignancy caused by the human T-cell lymphotropic virus type I (HTLV-1) with a very high prevalence in tropical areas but only a few cases reported in China.^[1,2]^ Diagnosis of ATLL requires histopathology and immunophenotyping of tumors, peripheral blood cytology, or morphology and immunophenotyping of peripheral blood and HTLV-1 serology. ATLL is clinically diverse, and is characterized by generalized lymphadenopathy; skin lesions, hepatosplenomegaly; leukocytosis involving an increased numbers of abnormal petal-like nuclei in lymphocytes.^[3]^ Cutaneous involvement is observed in approximately half of ATLL patients.^[4]^ ATLL lesions were categorized into patch (6.7%), plaque (26.9%), multipapular (19.3%), nodulotumoral (38.7%), erythrodermic (4.2%), and purpuric (4.2%) types. Based on these features, ATLL patients are likely to be misdiagnosed as skin diseases at the initial diagnosis, and this can lead to delayed treatment. Additionally, ATLL remains difficult to cure and has an extremely poor prognosis despite advances in chemotherapy and allogeneic hematopoietic stem cell transplantation. Delays in the diagnosis of ATLL can have an adverse effect on patients survival; thus, a greater awareness of this disease is necessary among physicians, specifically in primary hospitals in China.

The immune system is capable of recognizing and elimilating tumors. Therefore, elucidating the immune regulation mechanism of ATLL patients is particularly important for improving the survival rate of ATLL patients. The negative regulatory programmed death-1/programmed death-1 ligand 1 (PD-1/PD-L1) pathway has been implicated in the induction of cytotoxic T-lymphocyte (CTL) exhaustion during chronic viral infection, along with tumor escape from host immunity. Therefore, it is important to investigate the immunoregulatory receptor and costimulatory molecules on CD4^+^ ATLL cells.

In this report, we describe a rare case of ATLL in China with a focus on the cutaneous presentation, in order to shed light on the diagnostic indicators of ATLL in this population. Additionally, we examined the expression of immunoregulatory molecules on CD4^+^ ATLL cells in order to shed light on the immune-related effects of this lymphoma.

## 2 Case report

**Figure 1:**
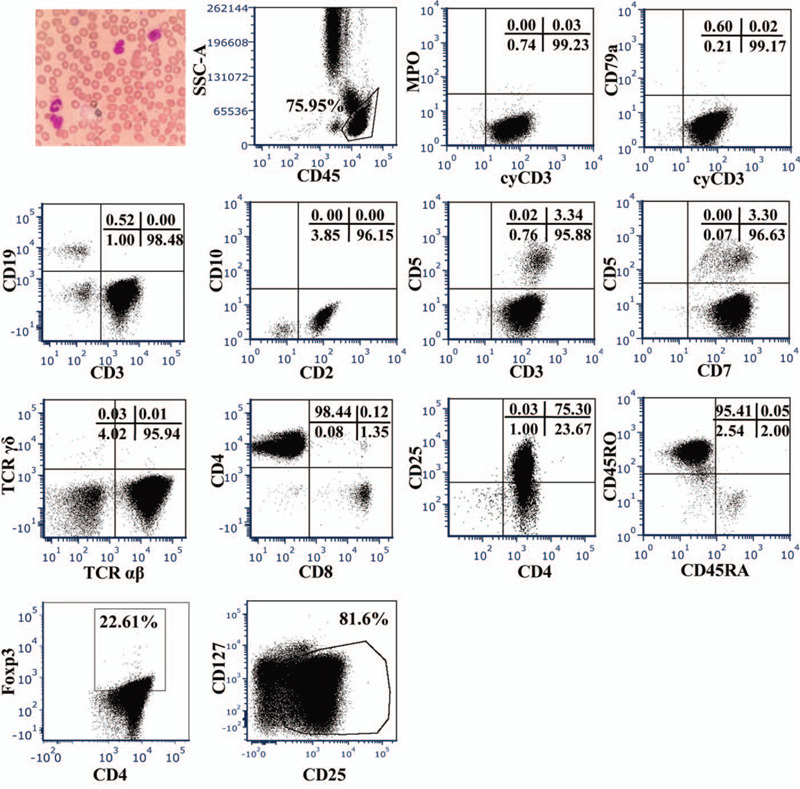
Peripheral blood smears and flow cytometric analysis of cyCD3, MPO, CD79a, CD3, CD19, CD5, CD2, CD7, CD4, CD8, TCRαβ, TCRγδ, CD25, CD45RO, CD45RA, Treg, and Foxp3 expression in peripheral blood of the patient.

**Figure 2:**
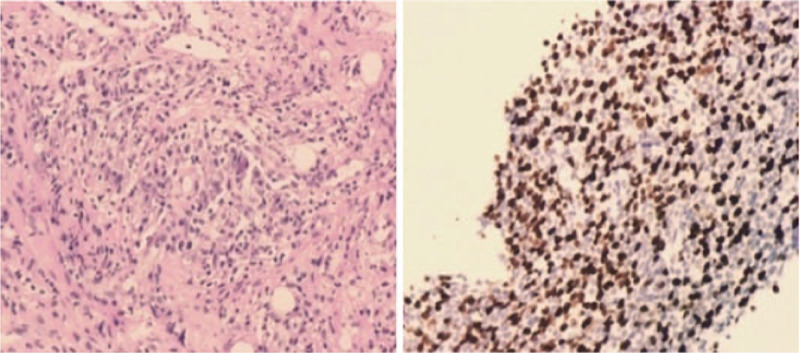
The tumor consisted of small- to medium-sized lymphoid cells with slightly fine nuclear chromatin (left, × 40); Almost all lymphoid cells were positive for CD3^+^ (right, × 40).

**Figure 3:**
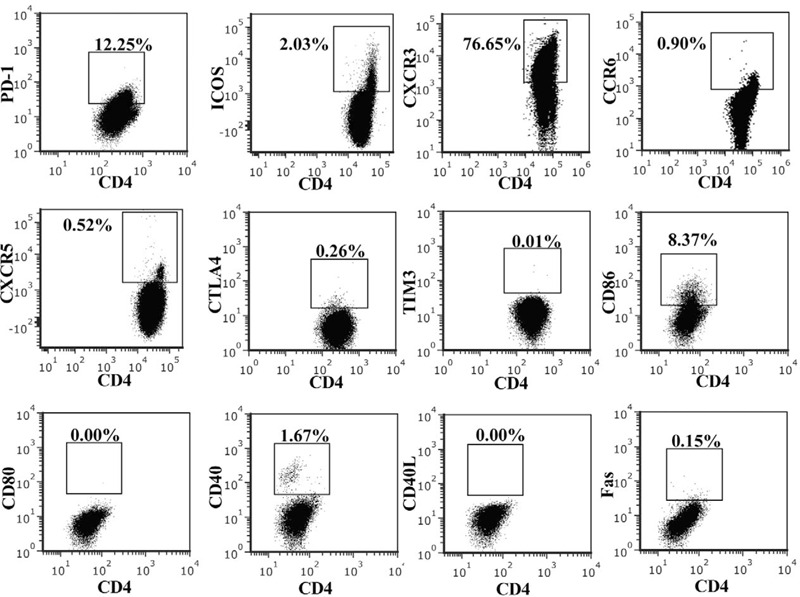
Flow cytometric analysis of PD-1, ICOS,CXCR3, CCR6, CXCR5, CTLA4, TIM3, CD86, CD80, CD40, CD40L, and Fas expression on CD4^+^ ATLL cells in peripheral blood of the patient.

A 51-year-old Chinese man was admitted to the hospital with a 2-years history of systemic skin plaque lesions and 1-year history of left ankle joint pain. Skin examination revealed erythema papules that were fused into patches. His laboratory tests revealed the following: white blood cell count, 27.50 × 10^9^/L (normal, 3.5–9.5 × 10^9^/L); lymphocytes count, 20.10 × 10^9^/L (normal, 1.1–3.2 × 10^9^/L); hemoglobin, 124 g/L (normal, 115–150 g/L); platelet count, 263 × 10^9^/L (normal, 125–350 × 10^9^/L); calcium 2.26 mmol/L (normal, 2.11–2.52 mmol/L); ferritin 565.8 μg/L (normal, 21.8–274.7 μg/L); immunoglobulin G, 18.90 g/L (normal, 7.51–15.60 g/L); lactate dehydrogenase, 244 U/L (normal, 120–250 U/L); high-sensitivity C-reactive protein, 66.4 mg/L (normal, 0–8 mg/L); erythrocyte sedimentation rate 71.9 mm/h (normal, 0–20 mm/hour); rheumatoid factor <20 IU/ml (normal, 0–20 IU/ml). Additionally, the patient was negative for antinuclear antibody, but serological examination revealed that he was positive for HTLV-1. CT examination revealed mediastinum and bilateral axillary lymphadenopathy that was indicative of malignant lymphoma (Stage IV). Peripheral blood smears showed pleomorphic, tortuous, petal-like nuclear lymphocytes (Fig. 1). Based on phenotypically examination, the neoplastic cells were classified as lymphocytes that express the pan-T-cell antigens cyCD3, CD3, CD2, and CD7 but lack CD5 (Fig. 1). The neoplastic cells (lymphocytes) were positive for CD45, TCR αβ, CD4, CD45RO, and CD25, and negative for MPO, CD79a, CD19, CD10, TCR γδ, and CD45RA (Fig. 1). These findings were indicative of ATLL. Lymph node needle core biopsy showed infiltration by atypical lymphocytes; this is consistent with the immunophenotype determined by flow cytometry (Fig. 2). We investigated the immunoregulatory receptors on CD4^+^ ATLL cells and found that CD4^+^ T cells expressed PD-1, inducible costimulator (ICOS), CXCR3, and CD86; partially expressed CCR6, and CXCR5 and did not express cytotoxic T-lymphocyteassociated antigen 4 (CTLA-4), T cell immunoglobulin domain and mucin domain-3 (TIM3), CD80, CD40, CD40L and Fas (Fig. 3). The patient received 3 cycles of cyclophosphamide, epirubicin/vinorelbine, dexamethasone (CHOP) chemotherapy. However, the tumor relapsed, and he underwent 1 cycle of epirubicin, vindesine, etoposide, and dexamethasone (EPOCH) chemotherapy. However, he did not respond to EPOCH chemotherapy. Therefore, his family discontinued treatment and opted for hospice care.

## 3 Discussion

In the present study, we present a case report on ATLL, which is rarely reported in China. The focus is on the cutaneous presentations and immunoregulatory molecules that could potentially be used for timely diagnosis and treatment.

ATLL, which appeared in southern Japan in 1976, is now common in Central and South America, Central Africa, the Middle East, far East, central Australia and Romania, but there has also been a few cases in China.^[1,2,4]^ ATLL is a rare non-Hodgkin's lymphoma. A common clinical manifestation is invasion of the skin by neoplastic cells that causes skin rashes of varying severity. Based on other case reports, the skin of the patients with ATLL could appear nodulotumoral, and exhibit plaques, maculae, purpura. Patients could also have multiple concomitant skin manifestations. In the present case, it was observed that the maculopapules on the face were fused with bilateral cheekbones, arch of the eyebrows, and neck, and the papules on his trunk had been itchy for 2 years.

ATLL is a peripheral mature T-lymphocyte malignancy that is etiologically associated with HTLV-1. ATLL is believed to originate from regulatory T cells (Tregs) based on the Treg phenotype, for example, expression of CD4, CD25, CD127, and Foxp3, as observed in our case.^[5]^ In the present case, investigation of the immunoregulatory receptors on CD4^+^ ATLL cells showed that they express PD-1, ICOS, and CXCR3; partially express CCR6 and CXCR5; and lack CTLA4 and TIM3. Further studies are warranted to reveal the precise mechanism of immune regulation in ATLL. PD-1 is a protein receptor that is constitutively expressed by Tregs and is essential for their suppressive function.^[6]^ The interaction between PD-1 and PD-L1 plays a role in the escape of tumor cells from T-cell immunity and negatively affects the impaired CD8^+^T cells in antiviral infection.^[7]^ ICOS, which is expressed in some activated CD25^+^Foxp3^hi^ Treg subsets in peripheral blood, is essential for maintaining autoimmune tolerance.^[8]^ ICOS expression on human Treg cells is closely related to IL-10 and TGFβ production capacity.^[9]^ CTLA-4 and TIM3 are protein receptors specifically expressed by Tregs and are essential for their suppressive function.^[10,11]^ Studies found that ATLL patients with PD-1-positive cutaneous lesions have more advanced dermatological and histopathological patterns than those with PD-1-negative cutaneous lesions.^[12]^ CCR6 is a seven-transmembrane Gprotein-coupled receptor that is selectively expressed by Th17 cells and Tregs.^[13,14]^ CXCR3 and its specific ligands are closely related to tumor immunity, tumor metastasis and antiviral immunity.^[15,16]^ CXCR5 is the specific receptor for CXC chemokine that enables T cells to migrate to the lymph node B cell region and play a key role in tissue infiltration of lymphocytes.^[17–19]^ We investigated the immunoregulatory receptor on CD4^+^ ATLL cells and found that CD4^+^ T cells express PD-1, ICOS, CXCR3, partly expression CCR6, CXCR5, and lack CTLA4, TIM3. The upregulated expression of PD-1 and ICOS as well as the lack of costimulatory molecules in this case may promote the immune escape of pathogens, but whether the expression of ICOS and PD-1 on ATLL is bidirectionally regulated or synergistically stimulated remains to be studied. Thus, further studies are warranted to reveal the precise mechanism of immune regulation in ATLL.

Costimulatory molecules are necessary for the activation of T cells. The absence of costimulatory molecules will lead to insufficient activation of T cells and the dysfunction of T cells. We further investigated the expression of costimulatory molecules on CD4^+^ ATLL cells and found that CD4^+^ T cells only express CD86 and but lacked CD80, CD40, and CD40L. Fas, as a member of the death-inducing family of tumor necrosis factors (receptors with an intracellular death domain) can initiate the extrinsic apoptosis signaling pathway.^[20]^ In the present case, Fas expression was not detected in the CD4^+^ ATLL, this might be associated with a reduction in the apoptosis rate and may be one of the reasons for the long-term survival of ATLL cells.

The present patient with ATLL exhibited immunodeficiency with an extremely poor prognosis, even when chemotherapy is employed which was similar to that reported by Kozako T.^[21]^ Studies have found that ATLL patients with PD-1-positive cutaneous lesions have more advanced dermatological and histopathological patterns than those with PD-1-negative cutaneous lesions.^[21]^

Although the patch and plaque types of ATLL exhibit a better survival rate,^[22]^ the delay in the diagnosis of ATLL can negatively affect patients survival. Based on the findings in the present case with plaque presentation, we recommend that patients with petal-like nuclear lymphocytes in blood smears, a high CD4: CD8 ratio, strong CD25 expression and skin patches undergo HTLV-1 serology testing.

## Author contributions

QHY and LNQ contributed to the concept and design of the study and to the acquisition, analysis or interpretation of working data. CSL and QJ analyzed the patient's data. All authors read and approved the final manuscript.

**Software:** Qinhua Yu.

**Writing – original draft:** Chen Shuang Lei, Qian Jiang.

**Writing – review & editing:** liannv Qiu.
